# Acute Calculous Cholecystitis Associated with Leptospirosis: Which is the Emergency? A Case Report and Literature Review

**DOI:** 10.2478/jccm-2024-0033

**Published:** 2024-07-31

**Authors:** Renata Moriczi, Mircea Gabriel Muresan, Radu Neagoe, Daniela Sala, Arpad Torok, Tivadar Bara, Ioan Alexandru Balmos, Razvan Ion, Anca Meda Vasiesiu

**Affiliations:** George Emil Palade University of Medicine, Pharmacy, Science, and Technology of Targu Mures, Romania

**Keywords:** leptospirosis, acute cholecystitis, gallbladder stones, Weil's disease

## Abstract

**Introduction:**

Leptospirosis is a bacterium with a worldwide distribution and belongs to the group of zoonoses that can affect both humans and animals. Most cases of leptospirosis present as a mild, anicteric infection. However, a small percentage of cases develop Weil’s disease, characterized by bleeding and elevated levels of bilirubin and liver enzymes. It can also cause inflammation of the gallbladder. Acute acalculous cholecystitis has been described as a manifestation of leptospirosis in a small percentage of cases; however, no association between leptospirosis and acute acalculous cholecystitis has been found in the literature.

**Case presentation:**

In this report, we describe the case of a 66-year-old patient who presented to the emergency department with a clinical picture dominated by fever, an altered general condition, abdominal pain in the right hypochondrium, nausea, and repeated vomiting. Acute calculous cholecystitis was diagnosed based on clinical, laboratory, and imaging findings. During preoperative preparation, the patient exhibited signs of liver and renal failure with severe coagulation disorders. Obstructive jaundice was excluded after performing an abdominal ultrasound and computed tomography scan. The suspicion of leptospirosis was then raised, and appropriate treatment for the infection was initiated. The acute cholecystitis symptoms went into remission, and the patient had a favorable outcome. Surgery was postponed until the infection was treated entirely, and a re-evaluation of the patient’s condition was conducted six-week later.

**Conclusions:**

The icterohemorrhagic form of leptospirosis, Weil’s disease, can mimic acute cholecystitis, including the form with gallstones. Therefore, to ensure an accurate diagnosis, leptospirosis should be suspected if the patient has risk factors. However, the order of treatments is not strictly established and will depend on the clinical picture and the patient’s prognosis.

## Introduction

Leptospirosis is a common zoonotic infection with increasing prevalence, a high incidence in the tropics, and considerable morbidity and mortality. Infection is caused by a spirochete belonging to the Leptospira genus, Leptospiraceae family [1, 2]. Based on World Health Organization data, the global incidence of leptospirosis is 1.03 million cases per year, with up to 58,000 deaths [3]. The infection is typically a mild, self-limited, nonfatal febrile form (90%) but can lead to a severe, icterohemorrhagic form called Weil’s disease (10%) that results in multiorgan failure. The disease primarily affects the kidney and liver and is associated with a significant decrease in the number of platelets [4, 5]. Rarely, myocardial tissue, the lungs, gallbladder, nervous system, pancreas, or ophthalmic tissue may be affected due to generalized vasculitis [6].

In 2021, in Romania, the incidence of leptospirosis was 0.06 cases per 100.000 inhabitants [7]. In this report, we present a case of acute calculous cholecystitis in a patient diagnosed with a severe form of leptospirosis. Informed consent was obtained from the patient regarding the publication of his case.

## Case presentation

**Fig. 1.**
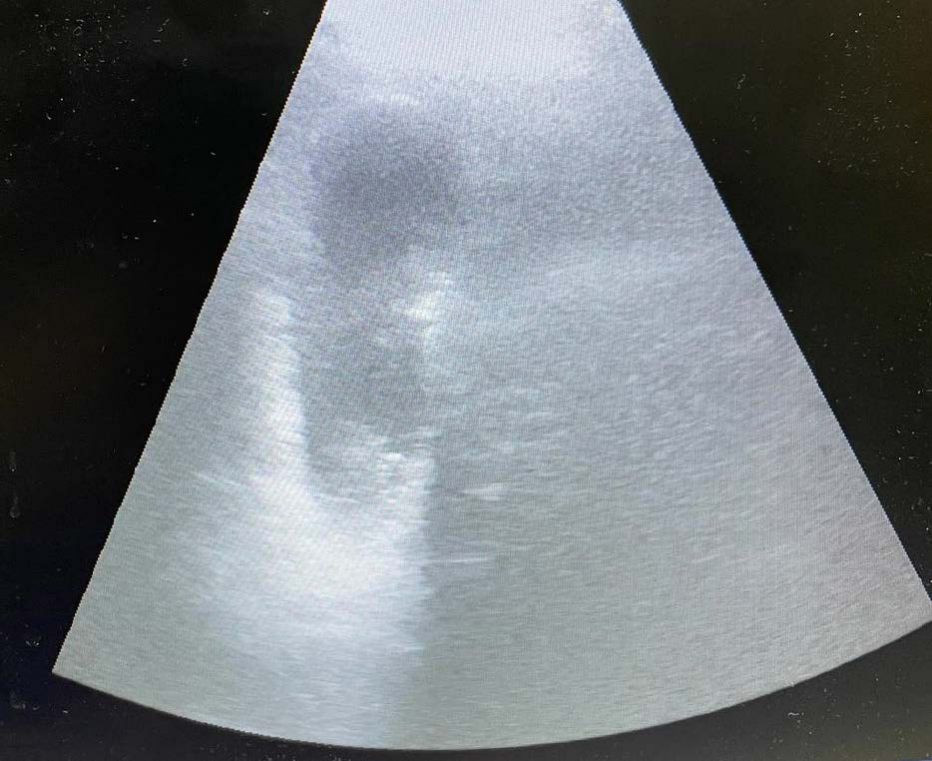
Acute calculous cholecystitis

A 66-year-old man with a prior history of arterial hypertension, chronic renal failure (G4 A1 stage), and type-2 diabetes presented himself to the emergency department with nausea, vomiting, fever, abdominal pain that was more pronounced in the right hypochondrium, and a four-day history of weakness. The patient denied recent travel abroad or known contact with infectious diseases. In the emergency department, biological samples were collected and blood cultures taken, with negative results for aerobic and anaerobic bacterial infections. An abdominal ultrasound (Figure 1) and computed tomography were performed, and a diagnosis of acute calculous cholecystitis was established.

**Fig. 2.**
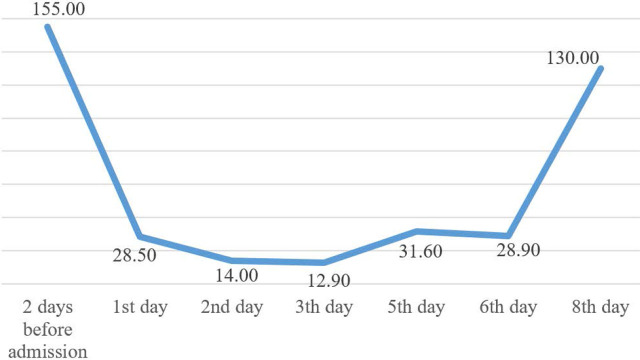
Platelet number (×10^3^/μL)

**Fig. 3.**
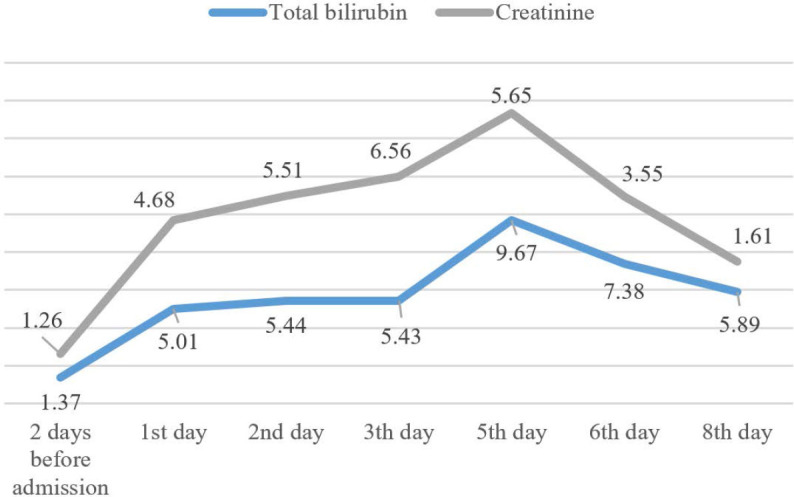
Total bilirubin and creatinine values during hospitalization (mg/dL)

The patient was admitted to the surgery department for preoperative preparation. Immediately after, he presented with severe thrombocytopenia, skin and scleral jaundice, subcutaneous suffusion, and signs of acute liver and renal failure. The surgery was postponed primarily because of the low platelet levels. The changes in the patient’s laboratory parameters are presented in Table 1, Figure 2 and 3.

**Table 1.** Changes in the patient’s laboratory parameters during hospitalization and six weeks after discharge

	**Two days before admission**	**Admission day**	**1^st^ day of treatment**	**2^nd^ day of treatment**	**4^th^ day of treatment**	**5^th^ day of treatment**	**8^th^ day of treatment**	**Six weeks after discharge**
Leucocyte (×10^3^/μL)	9.61	8.79	6.08	5.69	10.90	16.60	10.32	6.42
Neutrophils (%)	89.70	86.80	89	83	78	78.7	80.10	57.6
Lymphocyte (%)	6.61	6.63	0.27	9.40	0.98	8.34	1.10	34.3
Hemoglobin (g/dL)	14.10	12.4	10.40	10.80	12.60	14.00	12.3	14.2
Hematocrit (%)	42.60	36.4	30.10	31.90	37.80	41.50	34.7	44.6
Thrombocyte (×10^3^/μL)	155.00	28.5	14.00	12.90	31.60 ^*^	28.90 ^*^	130.00	241.00
AST (U/L)	25.00	75.00	78.00	123.00	48.00	45.60	91.80	24.00
ALT (U/L)	22.00	47.00	50.00	88.00	29.00	15.00	43.30	74.00
Total Bilirubin (mg/dL)	1.37	5.01	5.44	5.43	9.67	7.38	5.89	0.79
Direct Bilirubin (mg/dL)	-	4.14	-	-	-	7.13	4.69	0.29
C-Reactive Protein (mg/dL)	143.20	291.40	252.30	-	-	139.00	71.60	-
Urea (mg/dL)	34.24	115.56	128.40	186.18	196.88	169.00	104.00	53.00
Creatinine (mg/dL)	1.26	4.68	5.51	6.56	5.65	3.55	1.61	1.27
Calculated RFG (ml/min)	60.35	13.31	11.03	9.02	10.71	17.26	42.99	57.00
Amylase U/L	50.00	89.00	-	432.00	232.00	453.00	439.00	74.00

* the patient received one unit of platelet mass

Leptospirosis was suspected based on the patient’s clinical status, laboratory results, and history over the previous weeks, as he worked as an electrician in basements, with possible contact with rat urine. After confirming the diagnosis of leptospirosis (ELISA: immunoglobulin G antibody titer 9.647 DU, immunoglobulin M antibody titer 24.745 DU-positive for acute infection), antibiotic treatment was initiated with meropenem (3x1g/24 h) and vancomycin (1g/48 h) on the fifth day of his hospital admission.

**Fig. 4.**
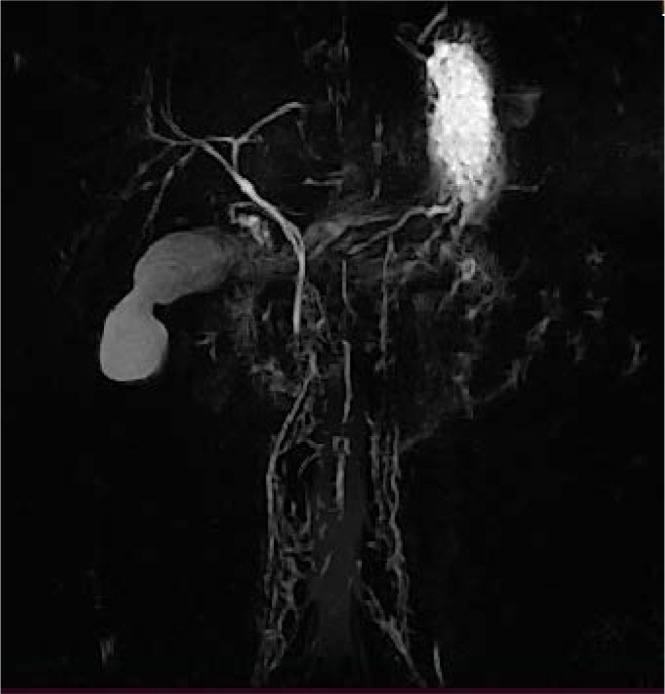
Magnetic resonance cholangiopancreatography (MRCP) imaging of the extrahepatic bile ducts

The acute cholecystitis resolved under symptomatic treatment. Serial imaging investigations (abdominal ultrasound and magnetic resonance cholangiopancreatography) showed a decrease in the pericholecystic inflammatory process, reduced thickness in the gallbladder wall, no dilation of the extrahepatic bile ducts, and no evidence of stones at the level of the common bile duct (Figure 4).

Directly after the leptospirosis diagnosis, the patient’s prognosis remained unfavorable. A pancreatic reaction had been observed, evidenced by a progressive increase in serum amylase, but without imaging or clinical signs of acute pancreatitis. Urea and creatinine values were increasing, coagulation imbalances could not be completely corrected, and the patient’s platelet count remained low. Therefore, the patient was transferred to the Department of Infectious Disease’s intensive care unit. Extensive supportive treatment of vital functions was initiated, restoring kidney and liver functions. A complex treatment regimen that included hydroelectrolytic rebalancing, antibiotics, gastric and liver protectors, parenteral nutrition, prophylactic anticoagulants, and antihistamine drugs was administered. During his hospitalization, a diagnosis of hepatitis C virus infection was also confirmed.

After 19 days of hospitalization and treatment, the patient was discharged in good general condition. He was afebrile, with hemodynamic and respiratory stability, and without abdominal pain. He exhibited normal renal functioning, anicteric skin, and normal laboratory parameters. A surgical re-evaluation was performed, and it was decided to postpone the cholecystectomy until the patient had achieved total recovery and remission of the leptospirosis, with a surgical re-evaluation scheduled six weeks later.

Six weeks after discharge, the patient presented to the hospital for a routine check-up in very good general condition, without subjective complaints. His renal and liver functions were within normal limits, coagulation and biochemical parameters were within normal limits, and he had normal complete blood count values (Table 1).

During this examination, an abdominal ultrasound was performed. The findings included abdominal meteorism, hepatic steatosis, gallbladder cholestasis with small stones, a cyst in the right kidney, atheromatosis of the abdominal aorta, and benign prostatic hypertrophy.

Due to the patient’s complete recovery from his Leptospira infection, the patient was then scheduled for elective laparoscopic cholecystectomy surgery.

We performed a literature search in the PubMed Central, Medline, Clarivate and Cochrane library database using the following search string: “leptospirosis and cholecystitis”. Patient who underwent abdominal imaging examinations (abdominal ultrasonography, or CT) were selected. Ten cases were described in the last decade in nine identified case reports [2, 4, 8,9,10,11,12,13,14]. The clinical and paraclinical results of these cases are summarized in Table 2.

**Table 2.** Ten cases of acute acalculous cholecystitis as a complication of leptospirosis [2, 4, 8,9,10,11,12,13,14]

**Author**	**V. Modesto dos Santos et al. [8]**	**P. Davies et al. [2]**	**D. A.R. Castelijn et al. [9]**	**D. A.R. Castelijn et al. [9]**	**H. Kilaru et al. [4]**	**N. Tepelenis et al. [11]**	**J. M. Villar et al. [12]**	**J. Vishalakshi et al. [13]**	**S. M.S Lim et al. [10]**	**H. Diktas et al. [14]**
Age	19	46	35	-	30	64	55	40	83	45
Sex	F	F	F	M	M	M	M	M	M	F
Country	Brazil	New Zealand	Netherlands	Netherlands	India	Greece	Spain	India	Malaysia	Turkey
Right hypochondrium or abdominal pain	Yes	Yes	Yes	Yes	yes	Yes	Yes	Yes	Yes	Yes
Jaundice	No	No	No	No	Yes	No	Yes	No	No	No
White blood cell number (×10^3^/μL)	7.7	2.0	10.90	4.4	ND	14.00	27.1	12.30	11.70	7.30
Total bilirubin (mg/dL)	ND	ND	48	34	2.1	3.1	12.4	3.9	0.91	0.3
IgM titer	ND	ND	ND	ND	Unequivocally positive	Strongly positive	Positive	Positive	Positive	Positive
Thrombocytopenia (/μL)	Yes (145,000)	Yes (84,000)	No (121,000)	No (121,000)	Yes (65,000)	Yes (95,000)	Yes (57,000)	Yes (85,000)	No (169,000)	No (316,000)
Imaging modalities	Abdominal ultrasound and CT	Abdominal ultrasound and MRCP	Abdominal ultrasound	Abdominal ultrasound	Abdominal ultrasound	Abdominal ultrasound and CT	CT	Abdominal ultrasound	CT	Abdominal ultrasound
Imaging findings for acute cholecystitis	Yes	Yes	Yes	Yes	No	Yes	Yes	Yes	Yes	Yes
Gallbladder stones	AAC	AAC	AAC	AAC	AAC	AAC	AAC	ACC	ACC	ACC
Renal failure	No	No	No	No	No	Yes	Yes	Yes	Yes	No
Associated pancreatitis or pancreatic reaction	No	No	no	No	No	No	No	No	Yes	No
Treatment	Antibiotics	Antibiotics	Antibiotics	Antibiotics	Antibiotics and open cholecystectomy	Antibiotics and open cholecystectomy	Antibiotics	Antibiotics	Antibiotics	Antibiotics
Complications	Pneumonia, Pericarditis	-	Pneumonia	Pneumonia	-	Chronic renal failure	Multiorgan failure, death	-	-	-

Abbreviations: AAC-Acute acalculous cholecystitis, CT-computed tomography, F-Female, M-Male, MRCP-magnetic resonance cholangiopancreatography, ND-Not done

## Discussion

Most cases of leptospirosis are self-limited and present a moderate or mild clinical infection without complications. In contrast, infections leading to jaundice, kidney failure, and hemorrhages, also known as Weil’s disease, can lead to long-term complications due to hematogenous dissemination. Determining the exact form of leptospirosis infection is critical because the therapy differs, and the mortality rate is strongly influenced by the patient’s symptoms and complications [15]. The mortality rate in the case of jaundiced patients can reach 19.1% in those with renal failure and from 12.1% up to 60% in patients over 60 years of age. However, in anicteric patients and those without complications, the mortality rate is nearly 0% [16].

Spirochetes enter the human body through abrasions, open wounds, or mucous membranes. Once they reach the bloodstream, they spread rapidly, affecting every organ, before the immune system can react and eliminate them via the host’s antibody response [17].

Leptospira can produce various symptoms that are not specific to the infection and depend on host and pathogen factors. The onset of symptoms typically occurs between 2 and 30 days after exposure, and the incubation time is 7 to 12 days [16]. These symptoms may include fever, chills, headache, and myalgia affecting the muscles of the back, thighs, and legs [17]. Gastrointestinal symptoms consist of abdominal pain, anorexia, nausea, vomiting, and diarrhea, and in approximately half of the cases, a nonproductive cough is present [18]. The suspicion of leptospirosis should be raised in patients who present with these symptoms and report a history of unsanitary working conditions or having direct contact with animals or their excrement. The more severe form of this infection, the icterohemorrhagic form, can have a much more rapid and severe course, with a high mortality rate without prompt and appropriate treatment [19].

A final diagnosis of leptospirosis can be established via cultures performed on Kortov’s medium, a polymerase chain reaction for DNA detection, or antibody detection with paired sera [20]. All of these methods take time before a result can be obtained. Therefore, for patients presenting with symptoms of renal and hepatic failure (i.e., an unexplained increase in serum bilirubin values without an obstruction in the bile ducts), initiating leptospirosis treatment is recommended as waiting for a confirmed diagnosis may lead to severe complications or death [21, 22].

Mild forms of leptospirosis do not require treatment. The antibiotics of choice for moderate cases include oral doxycycline, azithromycin, ampicillin, or amoxicillin [23, 24]. In severe cases, intravenous G penicillin was the treatment of choice but was ineffective in reducing the mortality rate or improving kidney function. In recent years, it has been replaced by doxycycline, ceftriaxone, or cefotaxime [25,26,27].

As a manifestation of leptospirosis infection, acute acalculous cholecystitis (AAC) occurs very rarely. Depending on the course of the infection, it may appear as an early (or late) manifestation of the disease. The pathogenesis underlying this association between the two conditions relates to the immune response against the leptospiras, which infiltrate the layers of the gallbladder wall. The leptospiras affect the blood vessels, causing vasculitis. Thus, infection can cause significant changes in the blood vessels in the muscular and serous layers of the gallbladder. Due to this damage, acute cholecystitis from leptospirosis can quickly lead to gangrene and perforation. Investigations into this scenario have revealed edema in the submucosa, mucosa loss, signs of tissue necrosis, suppurative inflammation, and endothelial damage in patients [4, 9].

AAC caused by Leptospira infection can progress to gangrene and even perforation, especially in patients where no specific risk factors for infection are present or there is no evidence-based suspicion. In instances where there is a delay in making the correct diagnosis or initiating the proper treatment, cases of AAC associated with leptospirosis have a much higher mortality rate (9.6%) than other causes of acute cholecystitis [4].

A real challenge is represented by the diagnosis of the obstructive icterus if at the ultrasound examination there are stones into the gallbladder, as it was in our case. Due do the inflammation occurred at the level of the gallbladder and the pancreas, the paralytic ileus, the ultrasound examination is limited in making the diagnosis in case of obstructive icterus and the location of the cause (stone, tumor). That’s the reason why, the gold standard investigation, if the level of urea and creatinine allow, is represented by the MRI cholangiography with a high accuracy in obstructive icterus diagnosis and also the location of the obstruction [28]. It was also our option in order to decide the treatment steps.

Studies have found that renal function will return to normal after the elimination of the infection. However, if renal failure is present, the mortality rate from leptospirosis is high [16, 29, 30]. Liver disease usually appears in patients with cholestasis, with significant increases in conjugated bilirubin and moderate increases in liver enzymes. In Weil’s disease, liver function slowly returns to normal, as the damage is reversible and does not influence the mortality rate [5, 31]. In the current case, the patient was already suffering chronic renal failure and cholestasis but without a known liver pathology. At the follow-up exam, six weeks after the infection, the patient’s renal function had not worsened despite acute renal failure superimposed on a chronic one.

While severe cases of leptospirosis can be complicated by AAC, no cases of acute calculous cholecystitis complicating leptospirosis were found in the literature. We have summarized the data obtained from published cases of leptospirosis and acute cholecystitis in the last decade (Table 2) and selected ten similar cases where the laboratory tests and the results of imaging investigations were presented.

We found that males were more prone to infection, probably due to their occupation and household activities [4, 9,10,11,12,13]. All patients complained of generalized or localized abdominal pain in the right hypochondrium upon presentation, but jaundice was present in the initial phase in only two cases [4, 12]. In nearly all patients, imaging signs of acute acalculous cholecystitis, such as split walls of the gallbladder or pericholecystic fluid, were described during the initial phase of infection. Thus, antibiotic treatment was initiated from the first hours of a patient’s presentation to a hospital and before obtaining a confirmed diagnosis of leptospirosis. The importance of imaging investigations should be emphasized, even in cases with non-specific symptoms, to assess the severity of the pathology and intervene with surgical treatment in complicated cases that do not adequately respond to conservative treatment. Only two patients required a cholecystectomy; both had a poor response to antibiotic therapy and presented signs of acute surgical abdomen and peritonitis [4, 11]. In some cases, AAC was associated with pancreatitis, and in severe cases, acute renal failure and thrombocytopenia were also present [2, 4, 8,9,10,11,12,13].

Leptospirosis has two phases: the first (acute and septic) lasts approximately one week, and the second (the immune phase) is dominated by the production of antibodies and the excretion of Leptospira in the urine. In this phase, the complication rate is the highest [21]. The long-term follow-up of these patients is essential to recognize possible complications, the most common being pneumonia. In our review, only one case resulted in multiple organ failure and death [21].

## Conclusion

The real novelty of the article consists in the association of the calculous cholecystitis with leptospirosis, previous renal and liver conditions, which it might have different and unpredicted evolution, treatment and complications. The progression of worsening biological parameters, especially the appearance of thrombocytopenia and signs of liver and kidney failure, led to a delay in planned surgery given the possible changes in organ functioning under general anesthesia and surgical shock. In addition to the association of vesicular lithiasis complicated with acute cholecystitis, the patient presented with a history of chronic renal failure and hepatitis C infection, both of which could have contributed to a complicated course of organ dysfunctions. Acute calculous cholecystitis is unlikely to result in complete remission without surgery. However, in our patient, the inflammation of the gallbladder walls was more likely the result of Weil’s syndrome, and it remitted after conservative treatment. This allowed us to schedule the surgery for the chronic calculous cholecystitis.

## Abbreviations

AAC: Acute acalculous cholecystitis
AST: Aspartate aminotransferase
ALT: Alanine aminotransferase
CT: computed tomography
DU: DRG units
IgG: Immunoglobulin G
IgM: Immunoglobulin M
MRCP: Magnetic resonance cholangiopancreatography
